# Effectiveness of Global Postural Reeducation in Postural Changes and Postural Stability in Young Adults

**DOI:** 10.3390/ijerph22010101

**Published:** 2025-01-13

**Authors:** Maria Paula Pacheco, Sara Morais, Paulo José Carvalho, Luís Cavalheiro, Filipa Sousa

**Affiliations:** 1 Coimbra Health School, Polytechnic University of Coimbra, 3046-854 Coimbra, Portugal; luisc@estescoimbra.pt; 2 Biomechanics Consultancy, 4000-122 Porto, Portugal; 3 RISE-Health|T.Bio, Polytechnic Institute of Porto, School of Health, 4200-072 Porto, Portugal; pmc@estsp.ipp.pt; 4 H&TRC—Health & Technology Research Center, Coimbra Health School, Polytechnic University of Coimbra, Rua 5 de Outubro, 3045-043 Coimbra, Portugal; 5 Porto Biomechanics Laboratory (LABIOMEP), University of Porto, 4200-450 Porto, Portugal; filipas@fade.up.pt; 6 Centre for Research, Education, Innovation, and Intervention in Sport (CIFI2D), Faculty of Sport of the University of Porto (FADEUP), 4200-450 Porto, Portugal

**Keywords:** Global Postural Reeducation, postural changes, postural stability, young adults

## Abstract

Background: Postural changes are considered a public health issue and have gathered significant interest in both research and clinical practice. Aims: To evaluate the effectiveness of Global Postural Reeducation (GPR) in improving postural changes and postural stability in healthy young adults. Additionally, this study aims to identify the main postural changes in the sample population. Methods: A longitudinal study was conducted with a sample of students (n = 38) from the 2nd and 3rd years of undergraduate programs at Coimbra Health School, divided into an experimental group (EG) with 20 subjects and a control group (CG) with 18 subjects. The EG underwent a GPR intervention, while the CG received no intervention. Postural changes were assessed using a 3D motion analysis system (Qualisys), and stabilometry was evaluated using a Bertec force platform. Results: At baseline (T0), the groups were homogeneous regarding sample characterization variables, as well as postural and stabilometric variables (*p* > 0.05). After four weeks of the intervention (T1), no significant differences were observed between the EG and CG for any of the variables studied (*p* > 0.05). However, within-group analysis for the experimental group revealed a significant difference (*p* = 0.04) in anterior-posterior velocity, indicating a reduction in this parameter from T0 to T1. In the control group, a significant difference was observed (*p* = 0.03) in the left knee valgus, indicating a reduction in valgus alignment. Conclusions: GPR does not appear to be effective in improving postural changes or center of pressure displacement in healthy young students.

## 1. Introduction

Posture can be described as any position adopted to maintain balance with maximal stability, utilizing minimal energy and avoiding overloading anatomical structures [1]. It results from a complex neuromotor and biomechanical system and is a dynamic, continuously evolving phenomenon. Posture corresponds to a body position maintained in space for a certain period, under the continuous control of the central nervous system (CNS), aiming to provide postural alignment and balance, thereby enabling the maintenance of a stable upright position against gravity [2,3]. The importance of quantifying posture has been widely emphasized in the literature. However, despite the availability of various methods for its assessment, each is related to specific strengths and limitations [4]. Optoelectronic motion capture systems are considered the gold standard for analyzing human movement [5]. On the other hand, maintaining posture while ensuring its stability is a fundamental task of the central nervous system (CNS) [6] for carrying out daily life activities, contributing to the well-being, independence, and quality of life of an adult human being [7]. The modern lifestyle associated with inadequate postural habits can contribute to early postural changes and subsequently to musculoskeletal symptoms, increasing the relevance of studying young and healthy populations [8,9]. It is established that upright posture is not completely static, with changes observed over short intervals of time when assessed through the center of pressure (COP) [10]. Parameters derived from COP trajectories, obtained using high-precision force platforms, are considered the gold standard for evaluating postural sway [11]. Postural changes are considered to be a public health issue and have gathered significant interest in both research and clinical practice [12]. Studies have revealed a prevalence of postural deviations ranging from 22% to 65% among children and adolescents aged 6 to 17 years [13,14]. In young adults (18 to 25 years), musculoskeletal pain symptoms are more prevalent in the neck, shoulders, and lumbar region, with percentages varying between 44.3% and 60.8%, 35.2% and 40.0%, and 46.8% and 50.2%, respectively [15,16]. Lumbar hyperlordosis [17,18], pelvic anteversion [17], genu valgum [19], forward head posture [20], and cervical hyperlordosis [21] have been described as the most frequent postural deviations. In students, one of the most common musculoskeletal issues is functional postural disorders [22]. A sedentary lifestyle is identified as a significant factor in the reduction of hamstring flexibility due to the shortening of muscles, tendons, and fascia resulting from long periods spent in a seated position [23]. The flexibility of the hamstring muscles is considered one of the main muscular influences of pelvic position. The stiffness of these muscles during spinal flexion can limit anterior pelvic tilt, exacerbating muscle and ligament tension in the lumbar region. This can, in turn, generate high compressive forces on the lumbar spine, compromising the anterior trunk flexion movement, a motion commonly present in daily life activities [24].

Global Postural Reeducation (GPR) is considered an innovative method of postural correction based on solid biomechanical and physiological concepts [25]. This method distinguishes dynamic muscles, activated in a phasic way, and static activated in a tonic way [26,27], and utilizes the viscoelastic properties of muscles, which allow for permanent stretching as long as the tension caused by the stretch of antigravity muscles is maintained for as long as possible, according to Hooke’s and Newton’s models [27,28]. The therapeutic approach of GPR is based on the principles of individuality and causality, as the real cause of the problem must be identified and treated, determining that the body should be handled as a whole [27].

The therapeutic approach is based on the integrated idea that the muscular system is composed of muscle chains located anteriorly and posteriorly to the vertebral column [29,30,31], consisting of several interconnected muscles with specific functional roles [29,32], which can shorten due to musculoskeletal changes, as well as anthropometric, behavioral, and psychological factors [27,32,33,34]. Its goal is to actively stretch the shortened muscles, reduce the pressure and align the joints, placing the entire body in maximal stretch, preventing adjustments, and increasing the contraction of the antagonist muscles to avoid postural asymmetries [29,33,34]. GPR employs a unique method for evaluating and diagnosing changes in the neuromusculoskeletal system. This approach utilizes specific postures that involve the dynamic adjustment of the hip joint angle, progressing from flexion to extension. The method is characterized by movements where the hips and knees transition through various angles, including hip flexion at 90°, followed by gradual knee extensions. These postures are specifically designed to stretch shortened muscles along the inspiratory, anterior, and posterior chains. [27,35]. This method is commonly known for treating postural changes [27,36], including scoliosis, cervical straightening, and lumbar hyperlordosis, among others, with the aim of rectifying the treatment of symptoms with a biomechanical origin [29,32,33,37,38,39], as well as the treatment of movement pattern disorders resulting from neurological conditions [40]. Several studies have demonstrated its effectiveness when compared to conventional physical therapy [41] in muscle flexibility [42,43,44], postural organization [42,45], functionality [33,40], cognition, quality of life [30,40,46], and pain [30,47]. The identification of postural changes through a thorough postural assessment is fundamental for diagnosing, planning, and monitoring the development of individuals during physiotherapeutic treatment [9]. The adoption of therapeutic measures for postural reeducation can be justified not only by the fact that poor postural alignment can change load distribution and pressure on joint surfaces, contributing to joint compression and inappropriate muscle tension but also by its potential role in prevention and health promotion, thereby contributing to the quality of life of individuals. This study aimed to evaluate the effectiveness of GPR in improving postural changes and postural stability in healthy young adults. It is also intended to identify the main postural changes of the sample elements.

## 2. Materials and Methods

### 2.1. Subjects and Experimental Procedure

This study was conducted in the physiotherapy laboratory of the Coimbra Health School at the Polytechnic University of Coimbra.

A prospective longitudinal study with a non-probabilistic sampling technique was selected from a previous study [16]. A random sampling technique was employed using the website www.randomizer.org for allocation into the experimental and control groups. Two assessment time points were considered. Initial moment (T0): before the start of the intervention; and Final moment (T1): after the therapeutic intervention process (4 weeks). Participants were recruited via email and phone contact from a pre-determined list, which included individuals who had previously participated in a study [16] and were classified as having postural changes. To be eligible, participants had to be enrolled in the 2nd or 3rd year of the undergraduate programs at the Coimbra Health School and present a positive Adams test, indicating trunk asymmetry when flexing forward, along with a thoracic hump of 5 degrees or greater, measured using a scoliometer. Exclusion criteria included the presence of any known musculoskeletal, neuromuscular, or cardiorespiratory pathology or injury; pain symptoms lasting more than 3 months; moderate to severe pain (VAS pain ≥ 5.0) at the time of assessment; and failure to sign the informed consent. The final sample consisted of 38 individuals, age 19.32 ± 1.19 yr, height 167.34 ± 10.04 cm, and body mass 63.75 ± 11.83 kg of both sexes participated in this study.

The variables under study were: (i) parameters involved in the assessment of postural changes proposed by the RPG method [27], where anatomical regions of the head, shoulders, thoracic spine, lumbar spine, pelvis, knees, and heels are observed, and when marked, correspond to the shortening of muscles that make up the inspiratory, anterior, and posterior chains; (ii) Vertical alignment of the body; (iii) Anterior trunk flexion; and (iv) Stabilometry.

The details regarding each of the variables are described in Table 1 and Table 2.

In the quantification of these postural changes, based on the angles and distances collected through the Qualysis system and described in Table 1, the following data were all considered normative values. For head position, a normal angle between 50° and 57° was considered [57,58,59]. A smaller angle was considered a forward head posture [48,60,61,62]. For the shoulders, the smaller the angle, the more anterior the shoulders are [48,60,63]. Angles below 52° represent rounded shoulders [59]. On the other hand, in the assessment of elevated shoulders, a positive angle indicates that the right shoulder is elevated, while a negative angle indicates the left shoulder is elevated [48]. The normal values for cervical lordosis and thoracic kyphosis range between 20° and 40° [63,64,65,66]. Lumbar lordosis is considered normal between 20° and 45° [67]. In the pelvis, the reference considers the positioning of the ASIS and PSIS in the same plane, corresponding to zero degrees [68]. However, a value of 12.26 ± 5.81° is acceptable [52]. Positive values indicate anterior pelvic tilt, while negative values correspond to posterior pelvic tilt [48]. Knee alignment corresponds to an angle of 180°. Alterations were considered for variations greater than 2.8° [69]. Angles greater than 180° correspond to varus, and angles less than 180° correspond to valgus [48]. For the calcaneus, values between 0° and 5° are considered normal [51]. Positive angles correspond to calcaneal valgus, and negative angles correspond to varus. To quantify the vertical alignment of the body, the angle defined by the intersection of the line connecting the acromion to the lateral malleolus with a vertical line was considered. An angle of 1.73 ± 0.94° was considered normal [52]. Trunk forward flexion was measured by the finger-to-floor distance, considering that the smaller the distance to the floor, the greater the spinal flexibility [70]. It is believed that the test represents the maximum length of the posterior muscle chain. Large distances between the fingers and toes, as well as increased angles at the ankle or hip, may indicate shortened muscles [71,72,73].

During data collection, participants were instructed to remain in a comfortable static position on the force platform, with their arms along the body, looking straight ahead, and with their forefeet abducted at a 30-degree angle [74]. The data collection for the standing position lasted 60 s, and participants were asked to position their upper limbs anatomically during the first 5 s. The duration of data collection for the forward trunk flexion was 30 s, and participants were asked to attempt to reach the floor with their fingers as much as possible, with their knees extended and arms and heads relaxed [75]. Data collection was performed once using ten high-speed infrared cameras from Qualisys Oqus 300, at a rate of 200 Hz, synchronized with a Bertec^®^ FP4060 force platform (Bertec Corporation, Columbus, OH, USA) with a sampling rate of 200 Hz. The capture and synchronization of kinetic and kinematic data were conducted using the Qualisys Track Manager (QTM) software, version 2.3. A total of 81 reflective markers were placed according to the protocol of the Istituto Ortopedico Rizzoli’s (IOR) [76] and the LABIOMEP Full Body Marker setup. The anatomical markers, the calculation of the postural variables of interest, and their interpretation are described in Table 1 and Table 2.

This study received approval from the Ethics Committee of the Faculty of Sport at the University of Porto (CEFADE 17.2019) on 17 July 2019 and was conducted in accordance with the Declaration of Helsinki.

### 2.2. Processing and Analysis of Data

The assessment and processing of kinematic data during the postural analysis were performed using the Qualisys Track Manager v2.15 software (Qualisys AB, Gothenburg, Sweden). The trajectory of each of the 81 markers used in the model was identified and labelled. The resulting three-dimensional (3D) data was exported to a tab-separated values (.tsv) file. A script was developed using R programming language (R version 4.2.2, Vienna, Austria) to process the .tsv file and calculate the variables described in Table 1.

Force plate data was also exported from QTM to a .tsv file, and analysed using the R “stabilo” package (version 0.1.1, https://doi.org/10.32614/CRAN.package.stabilo) [54]. Extracted variables are detailed in Table 2.

### 2.3. Intervention

**Figure 1:**
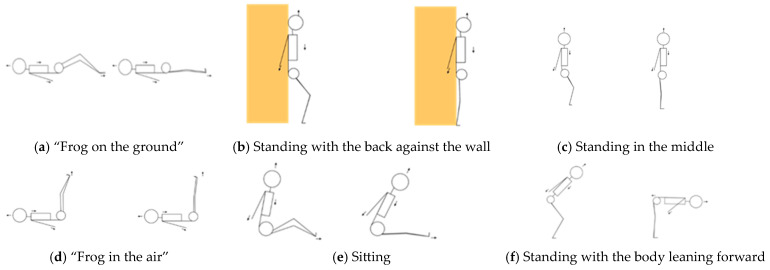
Global Postural Reeducation Postures. Arrows indicate direction of movement and orange color represents the wall.

The individuals in the experimental group underwent two weekly sessions of GPR, each lasting approximately 45 to 50 min. The sessions were conducted in groups consisting of 2 to 3 participants and were led by the same physiotherapist with experience in applying the method over a period of 4 weeks. The 6 GPR postures described by Souchard et al. [35] were applied, three of which involved opening the hip angle, characterized by a progression from flexion to the extension of hips and knees to stretch the inspiratory and anterior chain (“frog on the ground”, standing with the back against the wall, and standing in the middle) and three involved closing the hip angle, characterized by hips at 90° of flexion and performed gradual knee extensions to stretch the inspiratory and posterior chain (“frog in the air”, sitting, and standing with the body leaning forward) (Figure 1). Each posture was held for 15 min, except for the standing in the middle posture, which lasted 5 min. The progression process will depend greatly on the individuality of each participant. This means that the stretching was increased as the participant was able to maintain all postural corrections without compensations.

The description, details, and progression of the postures are presented in Table 3. All postures were performed with the intervention and supervision of the physiotherapist, involving manual contact to provide traction of the cervical and lumbar spine and to optimize the stretch while discouraging adjustments movements during the postures. The postures were also performed with the arms kept at a 45° abduction angle, with participants verbally instructed to adduct the scapulae, extend the arms toward the ground, and fully extend the elbows, wrists, and fingers.

The participants in the control group did not undergo any type of intervention.

### 2.4. Statistics

In the description and characterization of the sample, frequencies, as well as measures of central tendency and dispersion (mean and standard deviation), were used. For the comparison between groups, in the case of continuous variables, the student’s *t*-test for mean comparison was used, or its non-parametric counterpart, the Mann-Whitney U test. For dichotomous variables, the Chi-square test was used. For the analysis of the same group at different assessment points, the paired *t*-test was applied, specifically its non-parametric counterpart, the Wilcoxon test. The probability level accepted for statistical significance was *p* < 0.05.

## 3. Results

Table 4 highlights the general characteristics of the sample, demonstrating its homogeneity. No statistically significant differences were observed between the groups regarding age, weight, height, body mass index, and sex (*p* > 0.05).

From the analysis of the data related to the postural variables at baseline, it can be identified that the sample exhibits the main postural changes as forward head posture, rounded shoulders, cervical hyperlordosis, knee and calcaneal valgus, and lumbar hypolordosis (Table 5).

In the comparison between groups (experimental and control), no statistically significant differences were observed (*p* > 0.05), either at baseline or post-intervention (Table 5).

The observations vary in terms of the number of subjects, as it was not possible to collect data on all individuals and for all variables, as there are markers that would not be visible.

The results of the stabilometric variables are presented in Table 6. As with the previous variables, no statistically significant differences were found between the experimental group and the control group (*p* > 0.05), both at baseline and post-intervention (Table 6).

### Evolution T0 vs. T1

In the experimental group, when comparing T0 and T1, no statistically significant differences were observed (*p* > 0.05) in any of the postural variables. In the control group, only the variable ’Left Knee Valgus’ showed a significant difference (*p* = 0.033), indicating a decrease in knee valgus (Table 7). The observations vary in terms of the number of subjects, as it was not possible to collect data on all individuals and for all variables, as there are markers that would not be visible.

In the control group, when comparing T0 and T1, no statistically significant differences were observed (*p* > 0.05) in any of the center of pressure variables. In the experimental group, only the anteroposterior velocity variable showed a significant difference (*p* = 0.044), indicating a decrease in anteroposterior velocity from T0 to T1 (Table 8).

## 4. Discussion

This study aimed to evaluate the effectiveness of GPR on postural changes, trunk vertical alignment, overall spinal flexibility, and postural stability (center of pressure displacement) in young adults.

Postural changes were identified based on the examination of the retraining in Global Postural Reeducation, which evaluates its presence in the body segments of the head, cervical spine, shoulders, thoracic spine, lumbar spine, pelvis, knees, and calcaneus, corresponding to the shortening of the inspiratory, anterior, and posterior muscle chains. Through this analysis, it is possible to justify the identification of the main postural changes observed in our sample, showing a pattern of forward head and pelvic tilt, rounded shoulders, cervical hyperlordosis, and valgus knees and Calcaneus (Table 2). This pattern of changes is consistent with the results found in the literature, which report that forward head posture [20], rounded shoulders [77], cervical hyperlordosis [30], and valgus knees [28] are the most prevalent alterations. However, we found the result regarding lumbar lordosis in the control group to be unusual, as lumbar hyperlordosis is also reported in the literature as prevalent [17,18]. On the other hand, several authors note that the values corresponding to an adequate curvature are controversial, that the ideal range of lordosis remains unknown, and that it may be related to a variety of individual factors, such as weight, activity level, muscle strength, and flexibility of the spine and lower limbs [67,78]. Therefore, the normative values we considered may not be the most suitable for identifying lumbar curvature. The postural changes observed in our sample appear to primarily correspond, based on the examination of the retractions, to the shortening of the inspiratory and anterior chains [20]. We also evaluated the vertical alignment of the body and trunk flexion as global measures for assessing the retraction of the anterior and posterior chains. For vertical alignment and for trunk flexion, the values obtained by us are in accordance with the study by Krawczky et al. [52], which identified a reference value of 1.73 ± 0.94°, and with the study by Tulli et al. [79], which reported values of 20 ± 13.3 cm. Nevertheless, the observed values suggest a flexibility deficit, possibly indicative of posterior chain retraction. In this study, no statistically significant results were found between groups for any of the variables studied, namely postural changes, vertical alignment of the body, trunk anterior flexion, and center of pressure displacement (Table 5 and Table 6). Similar results regarding postural changes were reported by Cavalcanti et al. [80], who found that the ten GPR sessions were not sufficient to promote statistically significant improvements in the postural variables studied related to the body segments of the head, shoulders, thoracic kyphosis, and lumbar lordosis, either in the experimental group or in the control group, which did not undergo any intervention. In contrast to this, the study by Basso et al. [81] reported improvements in most of the postural variables assessed after ten GPR sessions, each lasting 45 min, conducted once a week with two postures per session. Similarly, Júnior and Tomaz [82] found that treatment with the RPG method was effective in postural correction, with improvements in head alignment and thoracic and lumbar scoliosis in nearly 50% of the 48 participants who underwent five GPR sessions over five consecutive days. Regarding trunk flexion, measured by the finger-to-floor distance, the results from the studies by Cavalcanti et al. [80] and Rosário et al. [83] differ from those found in this study. Both studies reported improvements in flexibility in GPR intervention programs conducted over 4 and 5 weeks, with the application of two postures per session. As for the results obtained in this study, the explanation for the discrepancies with other studies may lie in the number, duration, and frequency of the sessions, as well as the specificities of the intervention. Additionally, our sample consisted of young, healthy individuals with mild postural changes, which may have influenced the results. The same explanation can be applied to the results when comparing within the same group. We expected that, at least for the experimental group, there would have been some improvement in certain variables from T0 to T1 (Table 7 and Table 8). Although we found a statistically significant difference in the experimental group for the anteroposterior velocity variable (Table 8), this difference does not seem sufficient to guarantee the effectiveness of the intervention.

However, in the reviewed literature, studies by Lozano-Quijada et al. [8] and Fernandes et al. [34] were identified, which evaluated the effect of GPR on postural sway measured by the center of pressure (COP). In the first study, a single GPR session was conducted with university students, resulting in some modifications in postural sway, according to the authors. However, these should not be interpreted as improvements or changes in postural stability. The second study assessed the effects of GPR on women with chronic non-specific neck pain, focusing on pain, disability, and postural control. Eight biweekly sessions lasting 40 min over a 4-week period were conducted. The results of this study were statistically significant in all outcome measures except for postural stability. The anteroposterior velocity parameter in stabilometry can be considered, according to Massani et al. [84], an indicator of the efficiency of the postural control system and stands out as the most sensitive parameter. Lower values of the mean COP velocity are associated with better performance in postural control. Finally, the result with statistically significant differences observed in the control group for the postural variable left knee valgus (Table 7) does not seem particularly meaningful, especially considering that the values of evolution (from 176.37 ± 4.38 to 177.63 ± 3.10°) are not significantly divergent from the evolution observed in the experimental group (from 175.71 ± 3.44 to 176.53 ± 3.44°). This difference could be influenced by the sample size in each group. For the results obtained in the temporal evolution from T0 to T1 within each group (Table 7 and Table 8), in addition to the justification presented for the between-group comparison (Table 5 and Table 6), it seems that the fact that the intervention was conducted in a group setting, and therefore with less specificity and rigor in corrections tailored to each individual, may have contributed to the outcomes observed.

### Limitations

The main limitations of this study include certain characteristics of the sample, namely its relatively small size, as well as the fact that it consisted primarily of individuals without pain or identified pathology and with only mild postural changes. Although the primary objective was related to evaluating the effect of GPR on postural changes and considering, retrospectively, similar studies with different outcomes, we question whether the number of postures used (six), the session frequency (twice a week), and the intervention duration (four weeks) were optimal for achieving the intended goal. Additionally, the fact that the intervention was conducted in a group setting and thus was less personalized may have also contributed as a limitation. On the other hand, the normative data used to identify the presence of postural changes, which are not universally agreed upon in the literature, may represent another limitation. These norms, primarily determined through photogrammetric approaches or various measurement instruments, might not concretely reflect the differentiation between the presence or absence of postural changes. Secondarily, the identification of anatomical landmarks for applying markers in the use of the 3D motion analysis system, as well as the complex data processing required by this system, may introduce bias in data collection. This is particularly relevant as the procedure relies heavily on the training and anatomical knowledge of the evaluator(s). And the scarcity of studies evaluating the benefits of GPR in postural changes. In this context, there is a clear need for further research in this area involving larger, more diverse, and well-controlled samples across different populations, as well as more targeted and potentially more personalized interventions.

## 5. Conclusions

From the work conducted, we can conclude that, in our sample, it is possible to identify the presence of a pattern that indicates a tendency to a postural change in healthy young students, primarily characterized by anteriorization of the head and pelvis, rolled shoulders, cervical hyperlordosis, and valgus knees and calcaneus. Global Postural education does not appear to be effective in improving postural changes and center of pressure displacement in healthy young students. More studies will have to be carried out to better understand this theme.

## Figures and Tables

**Table 1:** Postural Variables, Anatomical Markers, and Description of Calculation.

Variables Under Study	Anatomical Markers	Calculation
Forward head posture [48]	Right Tragus (RTRAG); Spinous process of the 7th cervical vertebra (C7)	Angle C7—RTRAG with horizontal, in the sagittal plane.
Cervical lordosis [49]	RTRAG; and C7	Angle C7—RTRAG with vertical, in the sagittal plane.
Round shoulders [48]	Right and left acromion (RAC, LAC); and C7	Angle RAC, LAC—C7 with horizontal, in the sagittal plane.
Shoulders lifted [48]	RAC and LAC	Angle RAC—LAC with horizontal, in the frontal plane.
Thoracic kyphosis Adapted from [48]	C7, Spinous process of the 7th (T7) and 12th of thoracic vertebrae (T12)	Angle formed between the intersection of a line joining C7 to T7 and a line between T7 and T12
lumbar lordosis Adapted from [48]	T12; Third and fifth lumbar vertebrae (L3 L5)	Angle formed between the intersection of a line joining T12 to L3 and a line between L3 and L5
Pelvic tilt [48]	Right Anterior Superior Iliac Spine (RIAS) and Right Posterior Superior Iliac Spine (RIPS).	Angle formed between the intersection of a line joining RIPS and a projected line on a transverse plane at the level of RIAS joining RIAS and a point of the line projected perpendicularly from the plane to RIPS
Knee valgum/Varum [48,50]	Greater trochanter (RFT) Right/left lateral femoral epicondyle (RFLE/RFAL)	Angle formed between the intersection of the line joining RFT, RFLE and RFAL, in the frontal plane.
Calcaneus Valgus/Varus Adapted from [50,51]	Right/Left Posterior Surface of Calcaneus (RFCC/LFCC)	Angle formed between RFCC and LFCC a line parallel to the ground
Vertical alignment of the body [7,49,52]	RAC and RFAL	Angle formed between the line between RAC, RFAL and a vertical line drawn perpendicularly to the ground at the height of the lateral malleolus.
Distance from the right head of 2nd metacarpal (RMHC2) to the ground (cm) [50,53]	RMHC2	Distance from RMHC2 to the ground.

**Table 2:** Description of the stabilometric variables, based on the studies of Oliveira et al. and Prieto et al. [54,55,56].

Variables	Description	Interpretation
Area (cm^2^)	Area of a Stabilogram	Computes the area of a given statokinesiogram by fitting an ellipse containing 95% of statokinesiogram’s points.
sdx (cm)	Quantifies the Lateral Sway Amplitude	Calculation of the standard deviation of the lateral displacement of the center of pressure (COP) (variability)
sdy (cm)	Quantifies the anteroposterior Sway Amplitude	Calculation of the standard deviation of the anteroposterior displacement of the COP (variability)
xmdfreq (Hz)	Quantifies the Median Frequency of the Lateral Displacement of COP.	Computes the median frequency of the lateral displacement of the COP.
ymdfreq (Hz)	Quantifies the Median Frequency of the Anteroposterior Displacement of COP.	Compites the median frequency of the anteroposterior displacement of the COP.
xveloc (cm/s)	Mean lateral velocity of COP displacement	Computes the mean lateral velocity of a given COP displacement.
yveloc (cm/s)	Mean anteroposterior velocity of COP displacement	Computes the mean front-to-back velocity of a given COP displacement.

**Table 3:** Description, details, and progression of the postures.

Postures	Sessions	Description (Beginning of the Posture)	Progression
“Frog on the ground”,	1–5 and 7	Supine position with the lower limbs in flexion, abduction, and external rotation, with the soles of the feet together. Active expiratory correction of the thorax; manual traction of the cervical and lumbar regions during expiration with the aim of maintaining the physiological curvatures.	Fulfilled the condition acquired at the beginning of the posture, progressive extension of the hip with external rotation and adduction of the lower limbs, and dorsal flexion of the tibiotalar joint, growing the heels in the direction opposite to the head. Maintenance of self-growth, expiratory work stimulating the qualitative maintenance of the corrections.
Standing with the back against the wall	1, 2, 4, 6, 7 and 8	Standing with back against the wall, lower limbs in flexion, abduction, and external rotation, with the feet abducted at 30°, occipital traction and alignment in the vertical plane of the occiput, mid-dorsal region, and sacrum. Expiratory work, scapula adduction, and stretching of the upper limbs towards the ground.	Fulfilled the condition acquired at the beginning of the posture, progressive extension of the lower limbs with external rotation and adduction, active expiratory work. Maintenance of self-growth with the qualitative maintenance of the corrections.
Standing in the middle	1–8	A posture similar to the previous one, facilitating the integration of postural corrections under gravitational load.	Similar to the previous
“Frog in the air”,	1–3, and 6	Supine position with the lower limbs at 90 degrees of flexion, abduction, and external rotation of the thighs, with the soles of the feet together. Active expiratory correction of the thorax manual traction of the cervical and lumbar regions during expiration with the aim of maintaining the physiological curvatures.	Fulfilled the condition acquired at the beginning of the posture, progressive extension of the knees, keeping the sacrum on the ground, adduction and external rotation of the thighs, dorsiflexion of the ankles, and approximation of the medial malleoli with the feet abducted at 30°. Active expiratory work. Maintenance of self-growth with the qualitative maintenance of the corrections.
Sitting	2, 3, 5 and 7	Seated, supported on the ischial tuberosities, with the lower limbs in flexion, abduction, and external rotation, and the soles of the feet together. Active expiratory correction of the thorax, manual traction of the cervical region during expiration, aligning the occiput, mid-dorsal region, and sacrum in the same vertical plane.	Fulfilled the condition acquired at the beginning of the posture, progressive extension of the knees, avoiding lumbar kyphosis, adduction and external rotation of the thighs, dorsiflexion of the ankles, and approximation of the medial malleoli with the feet abducted at 30°. Active expiratory work. Maintenance of self-growth with the qualitative maintenance of the corrections. Gradual forward trunk flexion, slowly closing the hip angle.
Standing with the body leaning forward	4, 5, 6 and 8	Similar command to the previous posture, with the advantage of load on the lower limbs, which allows for a good stretch.	Similar to the previous one, but with load.

**Table 4:** Sample characterization.

Variables	Group	N	Mean	SD	*p* *
Age (years)	Experimental	20	19.50	1.28	0.320
	Control	18	19.11	1.08	
Weight (kg)	Experimental	20	66.50	11.12	0.133
	Control	18	60.70	12.16	
Height (cm)	Experimental	20	168.10	8.98	0.563
	Control	18	166.19	11.23	
BMI (kg/m^2^)	Experimental	20	23.51	3.38	0.070
	Control	18	21.77	2.14	
* *t* student test.					
		**Female**	**Male**	**Total**	*p* *
Sex	Experimental	12	8	20	0.671
	Control	12	6	18	
* Chi-square test.					

**Table 5:** Difference between groups in postural variables—T0 and T1.

	Baseline (T0)					Post Intervention (T1)				
Postural Variables/Degrees	Experimental Mean ± SD	n	Control Mean ± SD	n	*p* *	Experimental Mean ± SD	n	Control Mean ± SD	n	*p* *
Forward head posture	**44.97 ± 4.75**	19	**45.34 ± 6.78**	16	1.000	47.26 ± 5.11	17	45.96 ± 8.28	14	0.230
Right Round shoulder	55.71 ± 22.74	20	56.57 ± 18.35	18	0.828	54.50 ± 17.16	17	51.50 ± 18.57	14	0.625
Left Round shoulder	**36.47 ± 15.15**	20	**40.92 ± 16.21**	18	0.515	42.66 ± 19.07	17	40.29 ± 13.68	14	0.984
Thoracic kyphosis	26.94 ± 8.24	15	26.36 ± 6.92	18	0.442	28.69 ± 6.20	15	27.40 ± 6.52	13	0.751
Lumbar lordosis	25.33 ± 11.05	19	**17.86 ± 8.73**	18	0.053	23.34 ± 11.14	17	20.77 ± 11.47	13	0.621
Pelvic tilt	**10.98 ± 6.16**	19	**10.75 ± 5.25**	18	0.964	13.88 ± 7.46	17	11.22 ± 3.13	14	0.246
Right Knee valgum	**174.15 ± 2.60**	20	175.73 ± 3.93	18	0.303	175.42 ± 2.34	17	177.23 ± 4.44	13	0.094
Left Knee valgum	**175.29 ± 3.40**	20	**176.08 ± 3.08**	18	0.276	176.53 ± 2.15	17	177.63 ± 3.10	13	0.245
Right Calcaneus Valgus	4.83 ± 3.71	12	4.21 ± 3.31	10	0.821	3.96 ± 2.19	9	5.53 ± 3.63	7	0.606
Left Calcaneus Valgus	**5.12 ± 3.77**	15	**5.98 ± 4.33**	11	0.610	5.26 ± 3.08	11	5.99 ± 4.86	8	1.000
Cervical lordosis	**45.03 ± 4.75**	19	**44.66 ± 6.78**	16	1.000	42.74 ± 5.11	17	44.04 ± 8.28	14	0.230
Right Shoulder lifted	**1.61 ± 1.44**	9	**1.44 ± 1.09**	6	0.776	1.59 ± 1.37	6	1.19 ± 0.57	4	1.000
Left Shoulder lifted	**−1.53 ± 0.86**	11	**−1.97 ± 1.20**	12	0.566	−1.79 ± 1.23	11	−1.16 ± 0.83	10	0.251
Right Calcaneus Varus	−3.20 ± 2.78	7	−1.68 ± 1.28	8	0.336	−2.61 ± 1.73	8	−3.49 ± 1.96	7	0.536
Left Calcaneus Varus	−2.38 ± 1.84	4	−3.51 ± 3.51	7	0.648	−1.41 ± 1.09	6	−3.57 ± 2.12	6	0.093
Vertical alignment of body	1.63 ± 1.09	15	1.62 ± 1.07	18	0.873	1.03 ± 0.86	16	1.67 ± 1.02	13	0.092
RHMC2 to floor (cm)	24.79 ± 10.05	20	22.61 ± 9.27	18	0.762	24.67 ± 11.05	17	25.67 ± 13.17	14	0.681

* Mann-Whitney U test; Highlighted in **bold**—does not meet normative values defined (represents postural changes).

**Table 6:** Difference between groups in the center of pressure variables—T0 and T1.

	Baseline (T0)					Post Intervention (T1)				
Stabilometry Variables	Experimental Mean ± SD	n	Control Mean ± SD	n	*p* *	Experimental Mean ± SD	n	Control Mean ± SD	n	*p* *
Area (cm^2^)	1.37 ± 0.90	19	1.43 ± 0.68	18	0.599	1.29 ± 0.62	17	1.33 ± 0.43	14	0.570
sdx (cm)	0.36 ± 0.17	19	0.36 ± 0.11	18	0.558	37 ± 0.14	17	0.34 ± 0.06	14	0.769
sdy (cm)	0.20 ± 0.04	19	0.21 ± 0.06	18	0.461	0.19 ± 0.04	17	0.21 ± 0.05	14	0.230
xmdfreq (Hz)	0.22 ± 0.07	19	0.22 ± 0.07	18	0.893	0.22 ± 0.07	17	0.22 ± 0.06	14	0.953
ymdfreq (Hz)	0.33 ± 0.11	19	0.36 ± 0.13	18	0.343	0.36 ± 0.12	17	0.34 ± 0.11	14	0.953
xveloc (cm/s)	14.90 ± 2.27	19	16.15 ± 3.17	18	0.298	14.46 ± 2.40	17	15.05 ± 2.54	14	0.625
yveloc (cm/s)	21.80 ± 3.09	19	23.22 ± 4.46	18	0.538	21.03 ± 3.18	17	22.14 ± 3.73	14	0.518

sdx—Lateral Sway Amplitude; sdy—anteroposterior Sway Amplitude; xmdfreq—median frequency of the lateral displacement of COP; ymdfreq—median frequency of the anteroposterior displacement of COP; xveloc—mean lateral velocity of COP displacement; yveloc—Mean anteroposterior velocity of COP displacement. * Mann-Whitney U test.

**Table 7:** Comparison of postural variables at T0 and T1—Experimental and Control Groups.

	Experimental					Control				
Postural Variables/Degrees	Baseline (T0) Mean ± SD	n	Post Intervention (T1) Mean ± SD	n	p *	Baseline (T0) Mean ± SD	n	Post Intervention (T1) Mean ± SD	n	p *
Forward head posture	45.56 ± 4.75	16	47.45 ± 5.22	16	0.179	46.31 ± 6.86	12	45.92 ± 8.91	12	0.239
Right Round shoulder	57.29 ± 5.22	17	54.50 ± 17.16	17	0.309	54.66 ± 18.47	14	51.50 ± 18.57	14	0.300
Left Round shoulder	37.13 ± 16.34	17	42.66 ± 19.07	17	0.277	40.68 ± 17.59	14	40.29 ± 13.68	14	0.594
Thoracic kyphosis	26.62 ± 9.56	11	28.70 ± 6.38	11	0.594	26.56 ± 8.04	13	27.40 ± 6.52	13	0.463
Lumbar lordosis	26.16 ± 11.48	16	21.99 ± 11.48	16	0.278	18.07 ± 9.43	13	20.77 ± 11.47	13	0.279
Pelvic Tilt	10.57 ± 5.65	16	14.37 ± 7.42	16	0.056	10.65 ± 5.98	14	11.22 ± 3.13	14	0.875
Right Knee valgum	174.63 ± 2.38	17	175.42 ± 2.38	17	0.177	176.37 ± 4.38	13	177.23 ± 4.44	13	0.133
Left Knee valgum	175.71 ± 3.44	17	176.53 ± 3.44	17	0.177	176.49 ± 3.34	13	177.63 ± 3.10	13	0.033 ᵵ
Right Calcaneus Valgus	4.23 ± 3.95	5	4.36 ± 2.55	5	0.893	4.72 ± 4.57	5	4.99 ± 4.08	5	0.686
Left Calcaneus Valgus	4.76 ± 2.68	7	4.46 ± 1.85	7	0.866	7.22 ± 4.72	6	6.72 ± 5.46	6	0.917
Cervical lordosis	44.44 ± 4.75	16	42.55 ± 5.22	16	0.179	43.69 ± 6.86	12	44.08 ± 8.91	12	0.239
Right Shoulder lifted	1.86 ± 1.69	5	1.84 ± 1.37	5	0.893	1.87 ± 0.84	3	1.41 ± 0.43	3	0.285
Left Shoulder lifted	−1.73 ± 0.82	9	−1.91 ± 1.23	9	0.859	−2.07 ± 0.43	8	−1.28 ± 0.86	8	0.161
Right Calcaneus Varus	−2.77± 2.05	3	−3.13 ± 2.76	3	0.593	−0.98 ± 1.09	3	−3.44 ± 2.44	3	0.109
Left Calcaneus Varus	−0.10	1	−0.39	1	0.317	−4.25 ± 4.42	4	−4.13 ± 2.28	4	1.000
Vertical alignment of body	1.50 ± 0.96	12	1.29 ± 0.82	12	0.480	1.70 ± 1.13	13	1.67 ± 1.02	13	0.753
RHMC2 to floor (cm)	25.56 ± 10.35	17	24.67 ± 11.05	17	0.653	23.93 ± 9.55	14	25.67 ± 13.17	14	0.245

* Wilcoxon test; ᵵ *p* < 0.05.

**Table 8:** Comparison of center of pressure variables at T0 and T1—Experimental and Control Groups.

	Experimental					Control				
Stabilometry Variables	Baseline (T0) Mean ± SD	n	Post Intervention (T1) Mean ± SD	n	p *	Baseline (T0) Mean ± SD	n	Post Intervention (T1) Mean ± SD	n	p *
Area (cm^2^)	1.44 ± 0.95	16	1.30 ± 0.64	16	0.918	1.46 ± 0.67	14	1.33 ± 0.43	14	0.638
sdx (cm)	0.38 ± 0.18	16	0.37 ± 0.14	16	0.756	0.38 ± 0.11	14	0.34 ± 0.06	14	0.397
sdy (cm)	0.20 ± 0.04	16	0.19 ± 0.04	16	0.469	0.21 ± 0.05	14	0.21 ± 0.05	14	0.363
xmdfreq (Hz)	0.20 ± 0.06	16	0.22 ± 0.06	16	0.379	0.21 ± 0.06	14	0.22 ± 0.06	14	0.875
ymdfreq (Hz)	0.33 ± 0.11	16	0.35 ± 0.13	16	0.569	0.39 ± 0.13	14	0.34 ± 0.11	14	0.272
xveloc (cm/s)	14.86 ± 2.41	16	14.65 ± 2.34	16	0.215	15.26 ± 2.63	14	15.05 ± 2.54	14	0.221
yveloc (cm/s)	21.70 ± 3.25	16	21.31 ± 3.04	16	0.044 ᵵ	21.90 ± 3.67	14	22.14 ± 3.73	14	0.331

sdx—Lateral Sway Amplitude; sdy—anteroposterior Sway Amplitude; xmdfreq—median frequency of the lateral displacement of COP; ymdfreq—median frequency of the anteroposterior displacement of COP; xveloc—mean lateral velocity of COP displacement; yveloc—Mean anteroposterior velocity of COP displacement. * Wilcoxon test; ᵵ *p* < 0.05.

## Data Availability

The data that support the findings of this study are available on request from the corresponding author. The data are not publicly available due to privacy and ethical restrictions.
